# Introducing Analytical Science Advances

**DOI:** 10.1002/ansa.20190005

**Published:** 2020-03-24

**Authors:** Dietrich A. Volmer, Paul Trevorrow

**Affiliations:** ^1^ Editor‐in‐Chief ASA Humboldt Universität zu Berlin Berlin Germany; ^2^ Executive Journals Editor Wiley Chichester UK

Today, we launch *Analytical Science Advances* (ASA), a new open access analytical chemistry journal that will cover all fundamental and applied aspects of the analytical sciences, including interdisciplinary and newly emerging areas. ASA will rapidly disseminate analytical chemistry papers, regardless of the scope or field of science.

As part of the Wiley publishing group, ASA will guarantee the same high publishing, scientific, and editorial standards that authors and readers expect from an established publisher. ASA uses the established ScholarOne submission platform as most other Wiley journals and is overseen by three very experienced academic editors (Sebastiaan Eeltink, Christoph Steinbeck, and Dietrich Volmer), who have been editing other analytical and bioinformatics journals for many years, as well as an experienced interdisciplinary editorial board of scientists.

Readers may wonder why we decided to launch a new analytical journal at this particular time. There are in fact several reasons for this decision. First, during the past several years, the analytical chemistry field seems to have seen a slight erosion of contributions, indicating a shift of submissions to more general science journals. This loss might have been triggered by scientists’ impact factor considerations, in particular in those countries that operate national research assessment schemes, for example, the United Kingdom and Australia. It might also be related to the slow adoption of open science models in chemistry in general (including analytical chemistry), forcing some authors to move away from established community journals. Second, the publishing scene is currently rapidly changing to an open access model of publishing and several countries (including Germany, Hungary, and Norway) have now established agreements with Wiley, where open access charges are covered through national agreements. Finally, a new journal offers the community the opportunity to shape the journal to their specific requirements. A journal for the community by the community. A transformative journal with topical breadth in the age of “openness” moving forward with data requirements – data descriptor article types.

So how ASA differ from other analytical journals? As mentioned above, ASA provides an open analytical science platform. ASA is an open access journal and is committed to data sharing and open data standards for reporting storage and exchange of analytical data by its authors. Authors of analytical chemistry manuscripts who currently seek open science publication in more general science journals are invited to submit to ASA, where they find the same open science options but also the vital peer‐ship of the analytical chemistry community. In more generalist journals, peer‐reviewers are spread over many disciplines, making it much more difficult for editors to locate reviewers with the necessary expertise, often resulting in sub‐standard peer‐review reports. ASA has three analytical chemists as editors who are part of the analytical community. Moreover, at ASA, authors can directly access the academic editors via email or telephone for input or questions about their articles, with no anonymous, intermediate handling person at the publisher.

Another service offered by ASA is its encouragement for article transfers from a wide range of Wiley analytical titles, empowering authors with a direct home for their research in the event that their topic lies outside the criteria of the target journal. This function enables authors more expedient routes to publication without the laborious task of repositioning the submission for an alternative title.

ASA is very much a community journal, as such we are looking to highlight the community's vast array of special interest groups through special issues, as well as by general contribution. We welcome suggestions for such issues and look forward to having the opportunity to promote these communities through this publication.

Diversity lies at the fundamental core of the journal's mission, while this is reflected in the scope of the journal, it also extends to the breadth of our advisory board that promotes broad geographic, gender, and career level representation.

We hope that authors and readers will embrace the new journal. We are always interested in your feedback and suggestions for the new journal.

